# MENIN inhibitor-based therapy in acute leukemia: latest updates from the 2024 ASH annual meeting

**DOI:** 10.1186/s40164-025-00668-x

**Published:** 2025-05-22

**Authors:** Jiewen Sun, Wenjuan Yu, Xiang Zhang

**Affiliations:** 1 Women’s Hospital, Zhejiang University School of Medicine, Hangzhou, 310006 Zhejiang PR China; 2 Institute of Genetics, Zhejiang University School of Medicine, Hangzhou, 310058 Zhejiang PR China; 3 Department of Hematology, the First Affiliated Hospital, Zhejiang University School of Medicine, Hangzhou, 310003 Zhejiang PR China

**Keywords:** MENIN inhibitor, Acute leukemia, The 2024 ASH annual meeting

## Abstract

Menin inhibitors (MENINis) represent a novel and promising class of therapeutic agents for acute leukemia (AL). AL subtypes driven by overexpressed *HOXA9/MEIS1*, such as those characterized by *KMT2A*-rearranged (*KMT2Ar*) or *NPM1*-mutated (*NPM1m*) AL, display sensitivity to MENINi. Consequently, approximately 40–50% of acute myeloid leukemia (AML) and 5–15% of acute lymphoblastic leukemia (ALL) patients may potentially benefit from MENINi-based therapy. At the 2024 ASH annual meeting, updated clinical data regarding monotherapy with MENINis in AL, including revumenib, bleximenib, enzomenib and BN104, were presented. Moreover, combination therapies based on MENINis were also reported to be highly effective in refractory/relapsed, or newly diagnosed *KMT2Ar*- and *NPM1m*-AML patients. Evidently, MENINis have demonstrated a considerable efficacy in *KMT2Ar*- and *NPM1m*-AML patients with a well-tolerance. Furthermore, the therapeutic effects of venetoclax plus azacitidine or "3 + 7" regimens were further enhanced by the addition of MENINis in *KMT2Ar*- and *NPM1m*-AML patients. Therefore, MENINis offer new therapeutic prospects for AML patients, particularly for those with high-risky and poor-prognostic on-target subtypes.

## To the editor

MENINis represent a novel and promising class of therapeutic agents for AL. It is widely recognized that *KMT2Ar*-/*NPM1m*-AL exhibits sensitivity to MENINi. In fact, additional subtypes driven by overexpressed *HOXA9/MEIS1* may also be potential candidates for MENINi treatment [1]. Recently, revumenib became the first MENINi approved by FDA for refractory/relapsed *KMT2Ar*-AL, marking a significant milestone in the translation of MENINi from bench to bed. This paper highlights updates on MENINi for AL treatment as presented at the 2024 ASH annual meeting.

### MENINi as monotherapy

To our knowledge, more than seven distinct types of MENINis, including revumenib (SNDX-5613), ziftomenib (KO-539), bleximenib (JNJ-75276617), enzomenib (DSP-5336), icovamenib (BMF-219), BN104, and HMPL-506, are currently undergoing clinical trials [2]. Moreover, a number of studies have updated their results this year (Table 1).

**Table 1:** Updates of MENINi monotherapy for refractory/relapsed AL treatment in the 2024 ASH annual meeting

Inhibitor	Phase	Registration	Disease^1^	Genetic subtypes	Efficacy outcomes						Safety profile	Ref
					Evaluated cases^2^	ORR	cCR	CR/CRh	mTTFR^3^ (months)	mDoR (months)		
Revumenib	2	NCT04065399	AML, ALL, MPAL	*KMT2Ar*	97	64% (62/97)	42% (41/97)	23% (22/97)	/	6.4 in CR/CRh	Grade ≥ 3 febrile neutropenia (39%), anemia (20%), thrombocytopenia (16%), DS (15%), neutropenia (15%), leukopenia (15%), QTc prolongation (13%)	[3]
Bleximenib	1	NCT04811560	AML, ALL, other AL	*KMT2Ar*,* NPM1m*	150 mg BID: 20 90/100 mg BID: 20 45 mg BID: 13	50% (10/20) 50% (10/20) 39% (5/13)	40% (8/20) 40% (8/20) 23% (3/13)	30% (6/20) 35% (7/20) 23% (3/13)	/ 1.0 /	/ 6.4 /	All grade DS (13%), neutropenia (12%), thrombocytopenia (11%), QTc prolongation (0.8%)	[4]
Enzomenib	1	NCT04988555	AML, ALL	*KMT2Ar*,* NPM1m*, Others	*KMT2Ar*: 22 *NPM1m*: 13 *CALM-AF10*: 1	59% (13/22) 54% (7/13) 100% (1/1)	23% (5/22) 23% (3/13) 100% (1/1)	/	1.0	/	All grade febrile neutropenia (22.2%), DS (11.1%), QTc prolongation (5.0%)	[5]
BN104	1/2	NCT06052813	AML	*KMT2Ar*,* NPM1m*,* NUP98r*	11	89% (8/9)	33% (3/9)	/	0.9	/	All grade febrile neutropenia (20%), DS (10%), QTc prolongation (10%)	[6]

^1^The type of disease for current enrolled patients;

^2^The number of patients whose efficacy was evaluable;

^3^The definition of mTTFR: median time to first response, in which “response” referred to objective response

In the AUGMENT-101 study, continued treatment and follow-up data were updated after the interim analysis [3]. 97 refractory/relapsed *KMT2Ar*-AL patients were treated with revumenib. The ORR, cCR, or CR/CRh was 64%, 42% or 23%, respectively. The mDoR for CR/CRh patients was 6.4 months. 34% of patients with therapeutic responses proceed to HSCT.

Bleximenib was evaluated in refractory/relapsed *KMT2Ar*-/*NPM1m*-AL patients [4]. The ORR, cCR or CR/CRh were 50% vs. 50% vs. 39%, 40% vs. 40% vs. 23%, or 30% vs. 35% vs. 23% in 150 mg BID vs. 90/100 mg BID vs. 45 mg BID group, respectively. The mDoR in 90/100 mg BID group was 6.4 months.

Enzomenib was investigated in refractory/relapsed AL patients with *KMT2Ar*,* NPM1m* or other *HOXA9/MEIS1* driven subtypes, and 36 patients receiving active doses were evaluated [5]. The ORR and CR/CRh were 59.1% and 22.7% in *KMT2Ar* patients, while the ORR and CR/CRh were 53.8% and 23.1% in *NPM1m* patients.

BN104, a novel non-covalent MENINi, was evaluated in refractory/relapsed AML patients [6]. The ORR or CR/CRh was 89% or 33% in 9 *KMT2Ar*/*NPM1m* patients, respectively.

In comparison to *NPM1m*-AL, the therapeutic options for re-induction in *KMT2Ar*-AL are limited. Notably, MENINis offered a novel and effective therapeutic choice for *KMT2Ar*-AL patients.

### MENINi-based combination therapy

The investigation of revumenib and ziftomenib, two leading MENINis in clinical trials, extended beyond monotherapy, and bleximenib followed. Reports have also emerged regarding the outcomes of their combination therapy in AML treatments (Table 2).

**Table 2:** Updates of MENINi-based combination therapy for AML treatment in the 2024 ASH annual meeting

Inhibitor	Combination	Phase	Registration	Indication	Genetic subtype	Efficacy outcomes							Ref
						Evaluated cases^1^	ORR	cCR	CR/CRh	mDoR	RFS	OS	
Revumenib	ASTX727, Venetoclax	1/2	NCT05360160	Refractory/ Relapsed	*KMT2Ar*,* NPM1m*,* NUP98r*	26	88% (23/26)	69% (18/26)	58% (15/26)	Not reached in CR/CRh	59% for 6-month	74% for 6-month	[7]
Ziftomenib	Azacitidine, Venetocalx	1a	NCT05735184	Refractory/ Relapsed	*KMT2Ar*,* NPM1m*	*NPM1m* 200 mg: 5 *NPM1m* 400 mg: 6 *KMT2Ar* 200 mg: 7 *KMT2Ar* 400 mg: 6	100% (5/5) 67% (4/6) 43% (3/7) 33% (2/6)	80% (4/5) 50% (3/6) 29% (2/7) 17% (1/6)	/	/	/	/	[8]
Ziftomenib	Cytarabine, Daunorubicin	1a	NCT05735184	Newly diagnosed	*KMT2Ar*, high risky *NPM1m*	*NPM1m* 200 mg: 8 *NPM1m* 400 mg: 7 *KMT2Ar* 200 mg: 10 *KMT2Ar* 400 mg: 8	/	100% (8/8) 86% (6/7) 90% (9/10) 63% (5/8)	/	/	/	/	[9]
Bleximenib	Cytarabine, Daunorubicin/Idarubicin	1b	NCT05453903	Newly diagnosed	*KMT2Ar*,* NPM1m*	14	93% (13/14)	/	86% (12/14)	/	/	/	[10]

^1^The number of patients whose efficacy was evaluable

### Refractory/relapsed AML

The SAVE regimen (revumenib[SNDX-5613], ASTX727 and venetoclax) was specifically designed for refractory/relapsed AML [7]. The ORR was 88%, with a CR/CRh of 58%. 12 patients proceeded to HSCT. As follow-up, the 6-month RFS was 59% and OS was 74%. The mDoR had not been reached in CR/CRh patients.

In the KOMET-007 study [8], ziftomenib plus VA regimen was administrated to refractory/relapsed *KMT2Ar*-*/NPM1m*-AML patients. For *NPM1m* patients, the ORR was 100% at 200 mg and 67% at 400 mg; the cCR was 80% at 200 mg and 50% at 400 mg. For *KMT2Ar* patients, the ORR was 43% at 200 mg and 33% at 400 mg; the cCR was 29% at 200 mg and 17% at 400 mg.

### ND AML

In the KOMET-007 study [9], ND high-risk *KMT2Ar*-/*NPM1m*-AML patients were treated with ziftomenib plus "3 + 7" regimen. For *NPM1m* patients, the cCR were 100% at 200 mg and 86% at 400 mg, respectively. For *KMT2Ar* patients, the cCRR were 90% at 200 mg and 63% at 400 mg, respectively.

Bleximenib plus "3 + 7" regimen was also investigated in ND *KMT2Ar*-/*NPM1m*-AML patients [10]. 14 patients achieved an ORR of 93% and a CR/CRh of 86%.

As indicated, the addition of VA potentially only enhanced the therapeutic responses of MENINis in *NPM1m*-AML rather than *KMT2Ar*-AML among refractory/relapsed patients [8]. In contrast, the "3 + 7" regimen plus MENINi showed a durable and high response in both *KMT2Ar*-/*NPM1m*-AML among ND patients [9].

MENINis were well-tolerant, with their majority of AEs being manageable. As reported, gastrointestinal symptoms and cytopenias emerged as the most prevalent AEs. However, two relatively uncommon yet clinically significant AEs, DS and QTc prolongation, demand heightened clinical vigilance due to their potentially profound implications for patient safety and treatment outcomes.

Highlights from the 2024 ASH annual meeting regarding MENINi treatment for AL primarily focused on the monotherapy of novel MENINis and MENINis-based combination therapies with VA or "3 + 7" regimen. Notably, the addition of MENINi to standard therapies further improved therapeutic responses of *KMT2Ar-/NPM1m-*AML patients. These findings established its critical therapeutic role and held significant promise for KMT2A-MENIN-dependent AL. In the future, MENINis-based combination therapy still needed to be optimized and personalized for different AL subtypes.

## Data Availability

No datasets were generated or analysed during the current study.

## Abbreviations

AL: Acute leukemia
AEs: Adverse effects
ALL: Acute lymphoblastic leukemia
AML: Acute myeloid leukemia
ASH: American Society of Hematology
cCR: Composite complete remission
CR/CRh: CR plus CR with partial hematologic recovery
DS: Differentiation syndrome
FDA: Food and Drug Administration
HSCT: Hematopoietic stem cell transplantation
KMT2Ar: KMT2A-rearranged
KMT2Ar-/NPM1m: KMT2A-rearranged and NPM1-mutated
MPAL: Mixed phenotype acute leukemia
mDoR: Median duration of response
MENINi: MENIN inhibitor
mTTFR: Median time to first response
NPM1m: NPM1-mutated
ND: Newly diagnosed
ORR: Overall response rate
OS: Overall survival
RFS: Relapse-free survival
VA: Venetoclax and azacitidine
